# EphA4 targeting agents protect motor neurons from cell death induced by amyotrophic lateral sclerosis -astrocytes

**DOI:** 10.1016/j.isci.2022.104877

**Published:** 2022-08-05

**Authors:** Cassandra Dennys, Carlo Baggio, Rochelle Rodrigo, Florence Roussel, Anna Kulinich, Sarah Heintzman, Ashley Fox, Stephen J. Kolb, Pamela J. Shaw, Iryna M. Ethell, Maurizio Pellecchia, Kathrin C. Meyer

**Affiliations:** 1 Center for Gene Therapy, Nationwide Children’s Hospital, 700 Children’s Drive Columbus, OH 43205, USA; 2 Division of Biomedical Sciences, School of Medicine, University of California Riverside, 900 University Avenue, Riverside, CA 92521, USA; 3 Department of Neurology, The Ohio State University Medical Center, Columbus, OH, USA; 4 Department of Biological Chemistry & Pharmacology, The Ohio State University Wexner Medical Center, Columbus, OH, USA; 5 Molecular, Cellular & Developmental Biology Graduate Program, The Ohio State University, Columbus, OH, USA; 6 Department of Neuroscience, The Ohio State University Wexner Medical Center, Columbus, OH, USA; 7 Academic Unit of Neurology, Sheffield Institute for Translational Neuroscience, University of Sheffield, Sheffield S10 2HQ, UK

**Keywords:** Medical biochemistry, Molecular biology, Neuroscience

## Abstract

Amyotrophic lateral sclerosis (ALS) is a degenerative disease that progressively destroys motor neurons (MNs). Earlier studies identified EphA4, a receptor tyrosine kinase, as a possible disease-modifying gene. The complex interplay between the EphA4 receptor and its ephrin ligands in motor neurons and astrocytes has not yet been fully elucidated and includes a putative pro-apoptotic activity of the unbound receptor compared to ephrin-bound receptor. We recently reported that astrocytes from patients with ALS induce cell death in co-cultured MNs. Here we found that first-generation synthetic EphA4 agonistic agent 123C4, effectively protected MNs when co-cultured with reactive astrocytes from patients with ALS from multiple subgroups (sALS and mutant SOD1). Newer generation and more potent EphA4 agonistic agents 150D4, 150E8, and 150E7 provided effective protection at a lower therapeutic dose. Combined, the data suggest that the development of EphA4 agonistic agents provides potentially a promising therapeutic strategy for patients with ALS.

## Highlights

• We report on potent and selective EphA4 agents targeting its ligand-binding domain
• We used a method that generates neuronal progenitor cells from patient fibroblasts
• The agents reverse motor neuron cell death are cellular models of patients with ALS
• Our EphA4 agonists can effectively prevent astrocyte-mediated motor neuron toxicity

## Graphical abstract

**Figure:**
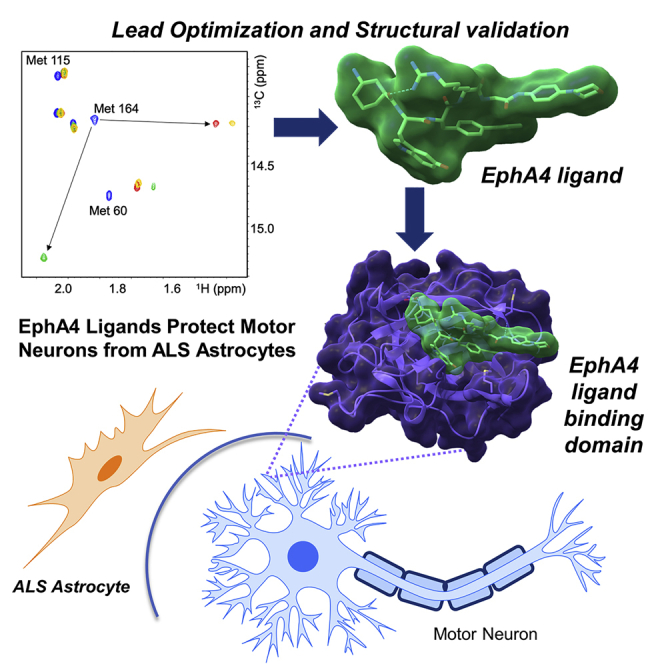

## Introduction

Amyotrophic lateral sclerosis (ALS) is a progressive degenerative motor neuron (MN) disease caused by both sporadic and genetic (familial) factors. Early studies identified several mutations in the antioxidant enzyme, SOD1 (superoxide dismutase 1), in the development of ALS (Rosen et al., 1993). Subsequent development of the first transgenic SOD1 mouse models indicates that mutations in SOD1 contribute to both onset and progression of ALS and has provided critical insight into underlying glial and motor neuron-mediated disease mechanisms (Bendotti and Carri, 2004; Nardo et al., 2016; Turner and Talbot, 2008). Consequently, most therapeutic strategies have utilized this mouse model for preclinical efficacy data. However, the use of SOD1 animal models is limited as most ALS cases develop owing to an unknown cause. Furthermore, SOD1 mutant mouse models do not represent additional disease mechanism caused by other gene mutations including TDP43, FUS, and C9ORF72 (Ajroud-Driss and Siddique, 2015; Baralle et al., 2013; Carri et al., 2006; Dejesus-Hernandez et al., 2011; Johann, 2017; Lagier-Tourenne and Cleveland, 2009; Orozco and Edbauer, 2013; Shenouda et al., 2018; Sreedharan, 2010; DeJesus-Hernandez et al., 2011). Thus, owing to the heterogeneity of patients with ALS (Ajroud-Driss and Siddique, 2015), successful translation of small molecule therapies to the clinic is limited.

To address this limitation, it is advisable to use both ALS animal models and patient genetic studies to aid in the discovery and validation of potential drug targets and therapeutics for ALS. For example, transgenic mutant-SOD1 zebrafish models identified the gene Rtk2, the genetic equivalent to the receptor tyrosine kinase EphA4 in humans, as a potential therapeutic target (Van Hoecke et al., 2012). This discovery was further corroborated by analyzing EphA4 expression levels in patients with ALS. A critical study demonstrated that EphA4 levels correlate inversely with both disease onset and overall survival. Furthermore, loss-of-function mutations in the EphA4 gene are associated with longer survival (Van Hoecke et al., 2012), suggesting that the modulation of EphA4 may be a promising therapeutic approach.

EphA4 binds to their natural ligands, the ephrins, inducing bidirectional signaling, and the ligands can interact with the receptor from the same cell or from adjacent cells (i.e. the astrocytes) (Pasquale, 2008). When EphA4 is aberrantly overexpressed, the unbound receptor can exert a pro-apoptotic activity in MNs, (Furne et al., 2009) whereas agonist bound receptors have no such cytotoxic effect. Thus, the modulation of ephrin activity through synthetic agonistic agents is potentially a viable therapeutic approach to protect MNs. Preliminary studies in the SOD1(G93A) mouse model demonstrated that the heterozygous deletion of the EphA4 gene before birth improved survival (Van Hoecke et al., 2012). However, follow-up studies showed that 50% ubiquitous reduction of EphA4 at symptom onset did not improve survival (Dominguez et al., 2020; Rue et al., 2019b; Zhao et al., 2018). This suggests that the reduction of EphA4 levels is not sufficient to provide a therapeutic benefit. Thus, an alternative therapeutic strategy would be to utilize ephrin-mimetics (agonistic agents) targeting its ligand-binding domain to ameliorate MN cell death induced by overexpression of EphA4 in patients with ALS with rapid disease progression. In agreement with this hypothesis, reducing ephrinA5 aggravated ALS disease progression, (Rue et al., 2019a) again perhaps suggesting that ephrinA5 mimetics could provide a benefit for patients with ALS.

Studies investigating ephrin mimetics were also independently evaluated as an alternative therapeutic approach to the genetic manipulation of EphA4 levels. These first studies utilized an EphA4 antagonistic peptide KYL (Murai et al., 2003) and showed delayed onset disease and improved survival in a rat model for ALS (Van Hoecke et al., 2012). However, the study also reported a similar *in vivo* effect with a previously reported small molecule agent (Noberini et al., 2008) that we and others (Tognolini et al., 2014; Wu et al., 2017) later recognized to be a false positive, and that hence did not target EphA4 potently nor specifically. An optimized and very potent EphA4 antagonistic cyclic peptide named APY-d3 (Olson et al., 2016) was also reported and studied *in vivo*. However, these studies suggested there was no significant difference between control and treated groups in both disease onset and survival (http://www.ephrins.org/doc/libro_abstract_2018.pdf). Similarly, a fusion protein combining the extracellular domain of wild-type EphA4 with an IgG Fc fragment (EphA4-Fc) has been proposed as a decoy to suppress EphA4 signaling. This agent has been shown to modestly improve survival but significantly delay disease onset in SOD1(G93A) mice models (Zhao et al., 2018).

Hence, our recent efforts focused on deriving potent and selective EphA4 agonistic agents, typified by first-generation EphA4 agonistic agent 123C4 (Table 1; Wu et al., 2017) We previously showed that 123C4 is moderately potent and selective toward the ligand-binding domain of EphA4 (K_d_ ∼ 400 nM, compared to ∼360 nM affinities reported by intact ephrin ligands such as ephrinA4 or ephrinA5 (Bowden et al., 2009a; Bowden et al., 2009b)). 123C4 acts as an agonist from the perspective of inducing EphA4 phosphorylation in primary cortical neurons, and improved survival, but not disease onset, in the SOD1(G93A) mouse model (Wu et al., 2017). More recently we reported on improved agonistic agents, including agent 150D4 (Baggio et al., 2021) and its new derivatives reported for the first time herein (Table 1). These agents were designed keeping in mind their predicted agonistic activity using biophysical approaches as reiterated later in discussion. (Baggio et al., 2021)

**Table 1:** Chemical structures and binding properties of selected EphA4 targeting agents

ID	Structure	MW	*K*_*d*_ (nM)^1^ vs EphA4	*K*_*d*_ (nM) vs EphA3	*K*_*d*_ (nM) vs EphA2	Aqueous solubility	Δδ Met 164	Δδ Met 60
123C4		807	425.4 ± 26.3 (n = 2)	>10,000	N.A.	∼100 μM	+	+
150D4		956	111.5 ± 1.4 (n = 2)	∼4000	N.A.	>50 mM	+++	++
150E7		969	128.7 ± 4.5 (n = 2)	ND	N.A.	>50 mM	++	+++
150E8		954	94.3 ± 4.4 (n = 2)	ND	N.A.	>50 mM	++++	++

Δδ values represent weight average perturbations observed in the ^1^H and ^13^C dimensions, as described in the methods. Δδ Met 164:; 0.1 ppm < + < 0.2 ppm; 0.2 ppm < ++ < 0.25 ppm; 0.25 ppm < +++ < 0.3 ppm; ++++ > 0.3 ppm. Δδ Met 60:; + < 0.05 ppm; 0.05 ppm < ++ < 0.1 ppm; +++ > 0.1 ppm. None of the agents induced a significant chemical shift perturbation to Met 115. N.A. = inactive under the experimental conditions. N.D. not determined. ^1^Mean ± SE are shown. The number of experiments is indicated in parentheses.

While our studies support EphA4 as a possible therapeutic target for patients with ALS with a G93A SOD1 mutation, the clinical effectiveness in human patients with different SOD1 mutations, or sporadic ALS development (sALS) has yet to be assessed. To investigate the therapeutic promise of EphA4 synthetic agonists in additional ALS backgrounds we utilized a rapid reprogramming method that generates neuronal progenitor cells (NPC) directly from patient fibroblasts. NPCs can subsequently be differentiated into astrocytes that are toxic to motor neurons. Here we use an astrocyte motor neuron co-culture to demonstrate the therapeutic effectiveness of 123C4, and newer generation EphA4 agonists such as 150D4, 150E7, and 150E8 in patients with ALS with either sporadic or mutant SOD1 forms of the disease (Table 1). We found that EphA4 agonists can effectively prevent astrocyte-mediated motor neuron toxicity *in vitro* in every patient cell line tested. Thus, we demonstrate the therapeutic potential of EphA4 agonists in multiple human ALS backgrounds suggesting promising therapeutic translation into a clinical setting. Perhaps most importantly, the agents we reported here represent invaluable pharmacological tools to further dissect the roles of EphA4 in ALS and potentially in several other human pathologies.

## Results

### Design and characterization of EphA4 agonistic agents

**Figure 1:**
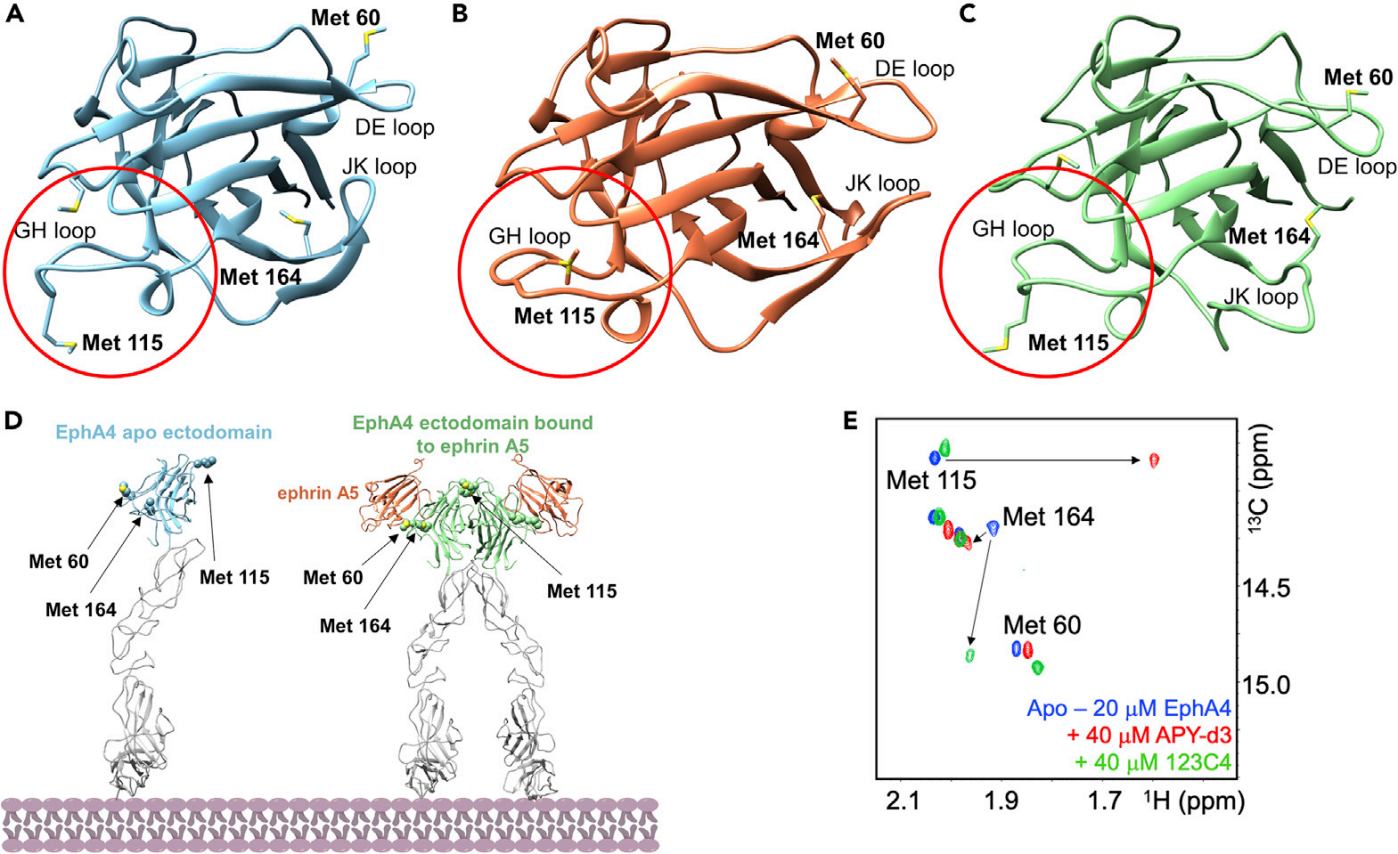
EphA4 receptor activation by agonistic ephrin ligands (A) X-ray structure of the ligand-binding domain (LBD) of EphA4 in its apo form (PDB ID 2WO1). (B) X-ray structure of EphA4 LBD bound to the antagonist agent APY-d3 (PDB ID 5JR2). (C) X-ray structure of EphA4 LBD bound to ephrin A5 (PDB ID 4BKA). In all three structures, key methionine residues and loop regions are highlighted. These are positioned within loops undergoing conformational rearrangement on agonistic (Met 164 in particular) or antagonistic (Met 115 in particular, in loop G-H highlighted with a red circle) binding. (D) Schematic representation of EphA4 receptor activation displaying the X-ray structure of the ectodomain of EphA4 in its apo form (PDB ID (4BK4), and the X-ray structure of the dimeric activated ectodomain of EphA4 bound to ephrin A5 (PDB ID 4BKA). Met residues present in critical loops involved in the conformational rearrangement on agonist binding are highlighted. Molecular models were analyzed using MOE 2019.0101 (Chemical Computing Group) or Chimera (www.cgl.ucsf.edu/chimera). (E) [^13^C,^1^H]- HSQC correlation spectra of ^13^C^ε^-Met labeled EphA4-LBD (29-209) (20 μM) in absence (blue) and in presence of 40 μM of APY-d3 or agonist 123C4. Note that the data reported in panel E) are essentially the same as we recently reported (Baggio et al., 2021) and are reiterated here simply to illustrate the strategy used to characterize 150D4, 150E7, and 150E8 (see Figure 2). Three critical Met residues assignments were obtained by single point mutations followed by ^13^C^ε^-Met labeling (Wu et al., 2017). Chemical shift changes occurred in a slow exchange in the NMR timescale as we previously reported (Baggio et al., 2021).

**Figure 2:**
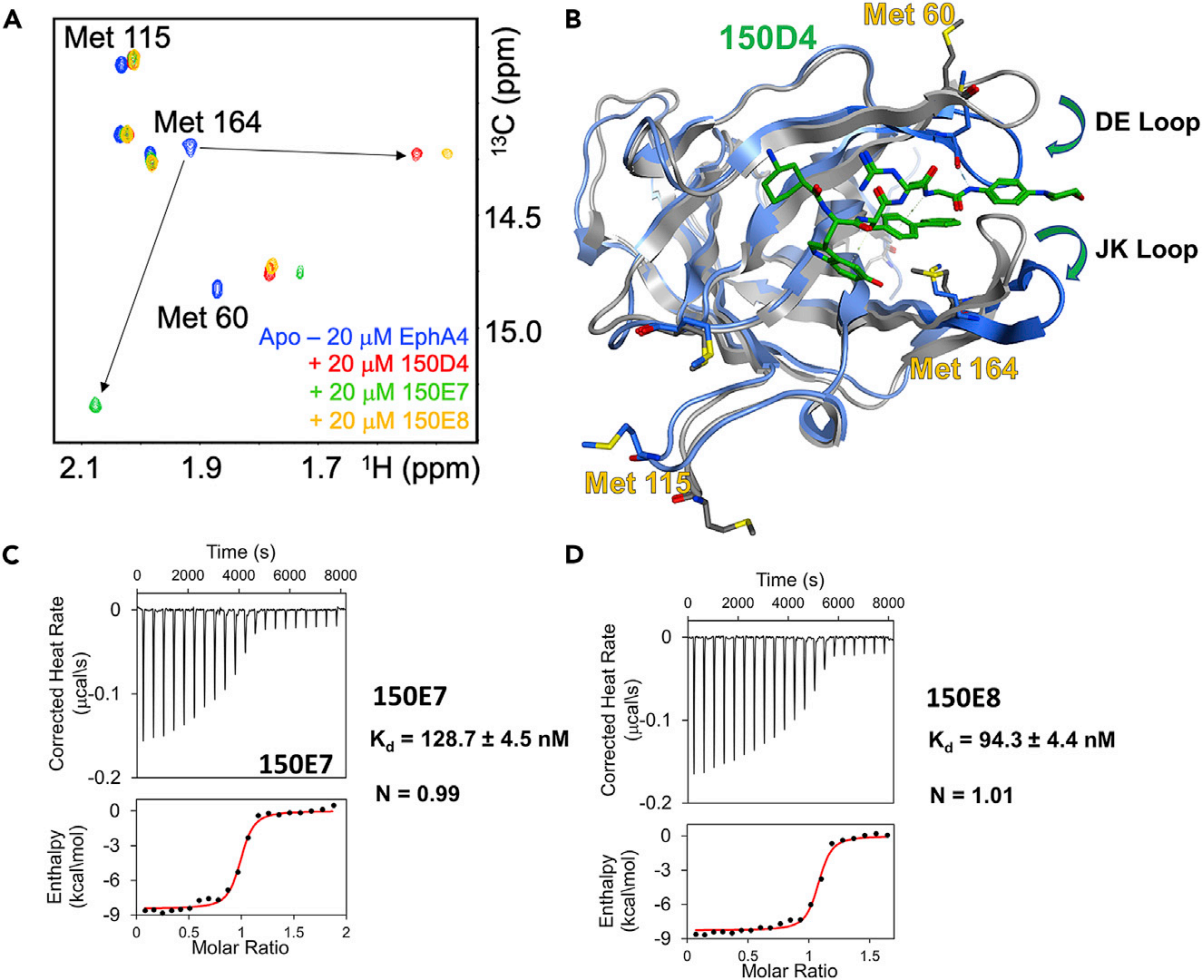
Biophysical characterizations of the binding of Table 1 ligands to EphA4 ligand-binding domain (A) 2D [^13^C, ^1^H] correlation spectra for EphA4-LBD ^13^C^ε^-Met labeled, measured in the absence and in presence of ligands 150D4, 150E7, or 150E8. Resonance assignments of these Met residues were obtained by single point mutations followed by ^13^C^ε^-methionine labeling and NMR analysis (Wu et al., 2017). (B) Superposition of the ribbon and stick model representing the X-ray structure of EphA4-LBD in complex with agonistic 150D4 agent (blue ribbon; PDB ID 7OFV) vs the apo structure of EphA4-LBD in gray (PDB ID 2WO1). Most notable conformational changes in agonist or antagonist binding are highlighted (loops JK, and DE, where Met 60 and Met 164 are located, show larger changes in agonist binding; on the contrary, Met 115 remains solvent exposed on binding. (C and D) ITC curves for the binding of new agents 150E7 or 150E8 to EphA4-LBD.

Previously, we deployed the HTS by NMR (High-throughput Screening by Nuclear Magnetic Resonance) approach targeting the EphA4 ligand-binding domain to identify possible ligands from a tri-peptide positional scanning library consisting of ∼125,000 agents (Wu et al., 2013). Initial screen and subsequent structure-activity relationships studies lead to agent 123C4 (Table 1), with an affinity for the isolated EphA4 ligand-binding domain (LBD) of ∼400 nM by isothermal titration calorimetry (ITC) (Wu et al., 2013, 2017). In follow-up studies, we derived a new combinatorial library based on the critical N-terminal aliphatic amine of 123C4 (a common feature also of antagonistic peptides) (Olson et al., 2016; Wu et al., 2013) and exploited our previous experience with the *f*HTS by NMR (Baggio et al., 2017), we rapidly identified a new hit molecule (Baggio et al., 2021). We also developed an efficient strategy to anticipate if ligand binding would induce changes in EphA4 conformation that are more compatible with agonist versus antagonist binding (Baggio et al., 2021). This was accomplished by protein NMR using EphA4-LBD that is uniformly labeled with ^13^C^ε^-methionine (Wu et al., 2017). Hence, 2D [^13^C,^1^H] correlation NMR spectra were measured in the absence or presence of test ligands and used to make qualitative determinations on possible conformational changes induced by test ligands on the protein target. In particular, EphA4 residues Met 164, Met 60 (in the binding site), and Met 115 are located in the D-E loop, J-K loop, and G-H loop of the EphA4-LBD, respectively (Figures 1A-1C). Structural studies indicate that the conformation of these loops is influenced differently by binding of an agonist (causing significant movements of loops J-K and D-E) versus an antagonist (that in particular seems to alter the position of Met 115 in the G-H but that does not alter significantly the other loops) (Figures 1B and 1C). Hence, monitoring chemical shift perturbations induced by test ligands to these Met residues provides a qualitative measure of the ability of the agents to act more as agonists or as antagonists. For example, we reported that the 13mer antagonistic cyclic peptide APY-d3 (Olson et al., 2016) induces changes not only in binding site Met 164 and Met 60 resonances, but also in Met 115, located in the G-H loop, that is at the EphA4-LBD dimerization interface (Figure 1D). Structural studies with APY-d3 (antagonist) or ephrin (agonist) bound EphA4-LBD revealed that Met 115 within the G-H loop assumes a rather different conformation in the antagonist bound form (from solvent exposed to more buried within the domain and the ligand), but it remains solvent exposed in the agonist form (Baggio et al., 2021). Hence, chemical shift perturbations induced by test ligands to the resonances of Met 115 (in particular), Met 164 and Met 60 can be used speculatively to anticipate if a ligand can cause conformational changes similar to those induced by an agonist or by an antagonist (Figure 1E). With these considerations in mind, ligand 150D4 was previously derived as described in our recent studies (Table 1; Baggio et al., 2021). In addition, we also report two additional modifications of 150D4, namely agents 150E7 and 150E8, that based on NMR studies also do not alter loop G-H, while inducing large chemical shifts of Met 164 and Met 60 (Table 1, Figure 2). Dissociation constant determination was obtained via isothermal titration calorimetry (ITC) measurements (Table 1, Figure 2). Similarly, selectivity studies were performed via ITC and ligand-binding domain constructs representing the two most closely related Eph domains, namely EphA3 and EphA2 (Table 1). In addition to being more potent for EphA4 compared to 123C4, the new agents are also significantly more water soluble, as determined by ^1^H NMR (Table 1). These studies culminated in the identification of agonistic agents targeting the EphA4 ligand-binding domain.

**Figure 3:**
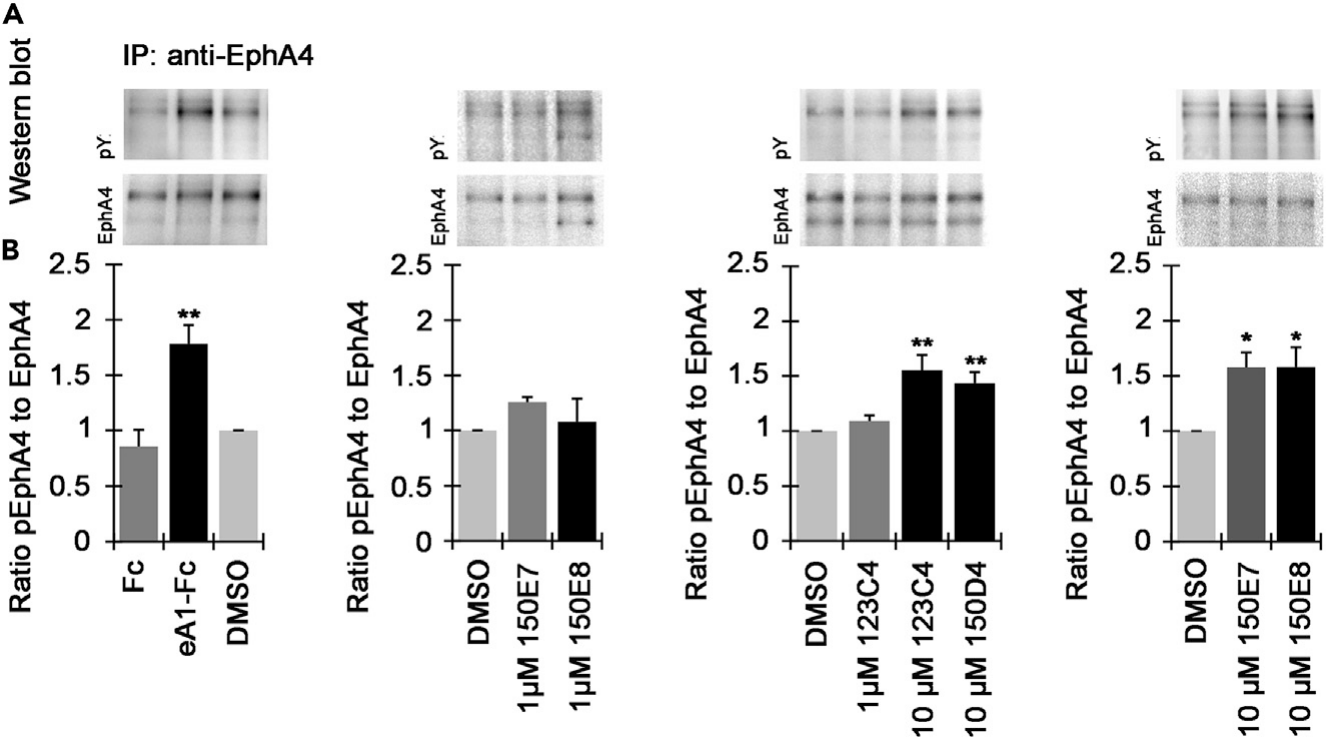
EphA4 phosphorylation in primary spinal cord motor neurons (A and B) Representative Western blot images of pEphA4 and total EphA4 (after immuno-precipitation, IP). (B) graphs showing an average ratio of pEphA4 to total EphA4 in cultures of primary spinal cord motor neurons treated with DMSO, Fc (2.5 μg/mL), ephrinA1-Fc (eA1-Fc, 2.5 μg/mL), 123C4, 150E7, 150E8 (1 and 10 μM) and 150D4 (10 μM) for 15 min. Each panel has its own controls and error bars indicate SEM (each experiment was repeated 3-4 times). Statistical analysis was performed using one-way ANOVA followed by Bonferroni’s post-hoc analysis (∗p < 0.05, ∗∗p < 0.01, compared to DMSO).

To verify that the agents can act as agonists in cellular assays we monitored the phosphorylation of its cytosolic kinase domain upon exposure to test ligands. For these determinations, we first isolated (postnatal day (P) 0-P2) primary motor neurons from mouse spinal cords (B_6_.Cg-Tg(Hlxb9-GFP)1Tmj/J (Hb9-GFP) mice). Subsequently, (2 days *in vitro* (DIV)) primary motor neurons were treated with pre-clustered Fc (2.5 μg/mL, as negative control), pre-clustered ephrinA1-Fc (2 μg/mL, as positive control), or 123C4, 150D4, 150 × 10^7^, or 150 × 10^8^, at various concentrations. Before processing for Western blotting, cells were treated for 15 min at 37 °C under 5% CO_2_/10% O_2_ atmosphere. To maximize ephrinA1-Fc activity, clustering was accomplished with goat anti-human IgG (Jackson ImmunoResearch, #109-005-003; 1 h at 4 °C). EphA4 phosphorylation was determined by the following procedure: lysates were exposed to protein-A agarose beads (Sigma, #P1406) and anti-EphA4 antibody (Invitrogen, #371600), for 2 h at 4 °C, and subsequently boiled in reducing conditions, spun down, and the supernatant was subjected to WB analysis with an anti-phosphotyrosine antibody and re-probed with EphA4 antibody (Figure 3). The data (Figure 3) confirm that the agents are capable to induce EphA4 phosphorylation similar to what was observed by clustered ephrinA1-Fc, although being qualitative in nature we do not seem to be able to discriminate between the activity of 123C4 and the newer agents in such assay. Hence, we studied the comparative effect of 123C4 and the new agents in more direct neuroprotection assays as described later in discussion.

### Activity of EphA4 agonistic agents on motor neurons

**Figure 4:**
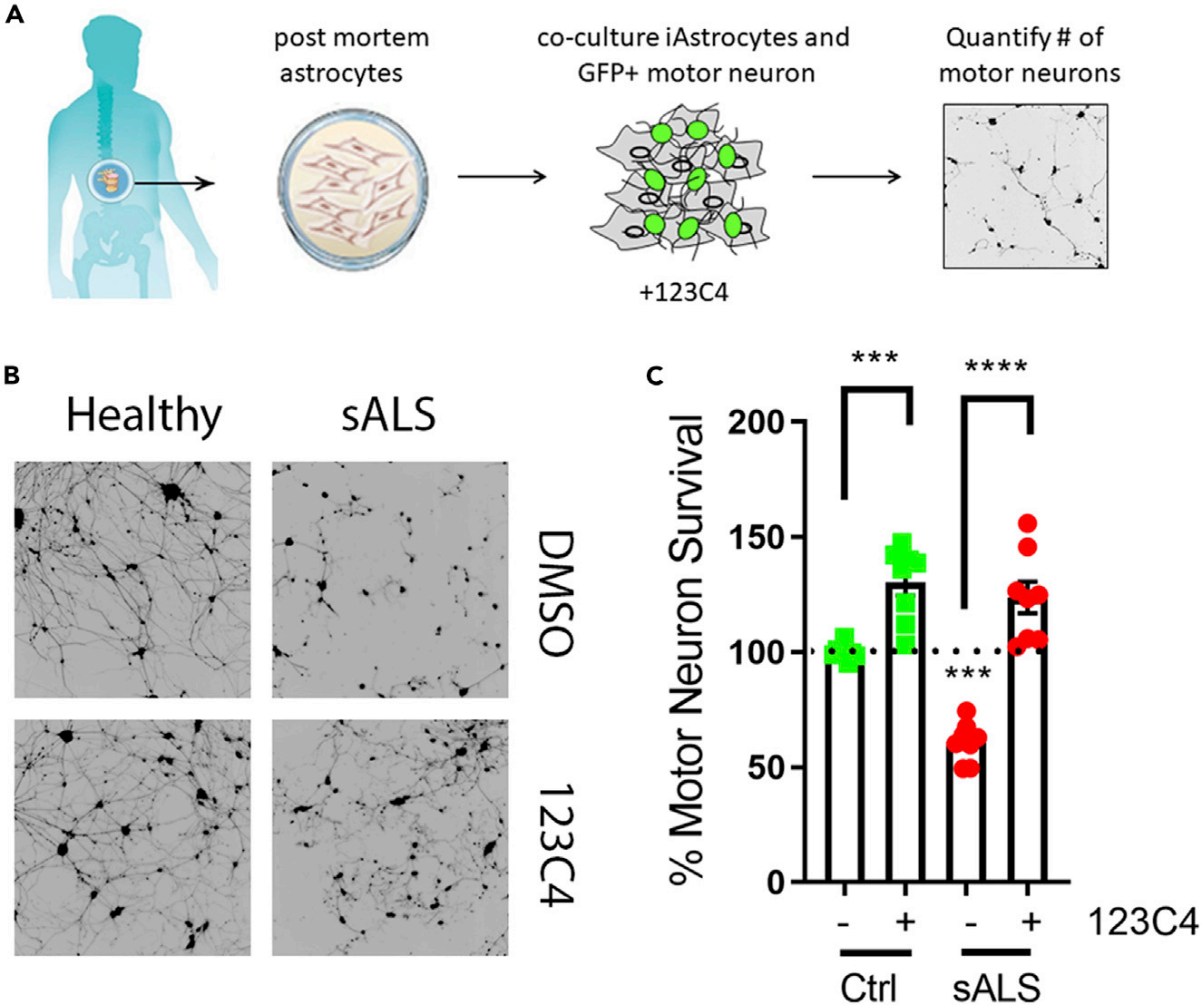
Ephrin Agonist 123C4 protects motor neurons from sALS astrocyte-mediated toxicity (A) Drug screen co-culture schematic. (B) Representative images of motor neurons (black) seed on top of astrocyte monolayer. (C) Quantification of motor neuron survival. Only motor neurons with neurites exceeding 50 microns were counted. 123C4 was added at 100 μM concentration at the time of motor neuron seeding. 123C4 was not removed from co-culture. Data represent three independent experiments utilizing separate motor neuron differentiations and different passages of postmortem-derived astrocytes. Data from each separate experiment were combined and statistical analysis was performed using Student’s *t* test against healthy control or corresponding untreated control. In addition, a separate T-test analysis was performed comparing treated cells to their corresponding untreated controls (line) (∗p < 0.05, ∗∗p < 0.005, ∗∗∗p < 0.0005, ∗∗∗∗p < 0.0001). The black signal in the images of motor neurons represents the Hb9-GFP.

Ephrin receptors are expressed both on astrocytes and motor neurons, regulating motor neuron cell death by preventing neuronal pro-apoptotic signaling from the monomeric form of the receptor (Furne et al., 2009). Thus, to assess the therapeutic potential of ephrin-mimetics in preventing motor neuron death we sought first to evaluate the effects of 123C4 (Table 1) on human astrocytes-mediated motor neuron toxicity. To do this, we obtained postmortem-derived human astrocytes directly from the spinal cord of a patient who died of sporadic ALS. Healthy astrocytes were obtained from a donor who died of non–disease-related causes. These cells were co-cultured with mouse motor neurons expressing GFP under the HB9 promotor as previously described (Haidet-Phillips et al., 2011). 123C4 was added to the co-culture system while the motor neurons were seeded (Figure 4A). Five days following the start of co-culture, motor neurons were imaged (Figure 4B) and the number of healthy neurons was quantified. The results indicated that astrocytes obtained from patients with sALS were toxic to motor neurons. Importantly, the addition of 123C4 at the time of motor neuron seeding prevented sALS astrocyte-mediated motor neuron toxicity (Figures 4B and 4C). Thus, these findings suggest that EphA4 agonist 123C4 can prevent non-cell autonomous-mediated cell death.

However, astrocytes obtained from postmortem samples more closely reflect disease end-stage, hence compound screening may not directly select agents that could benefit patients. Thus, we have previously developed a system to reprogram patient fibroblasts into induced neuronal progenitor cells (NPCs). NPCs can subsequently be differentiated into induced Astrocytes (iAs) supported by GFAP staining (supplementary Figure S3) (Dennys et al., 2021; Meyer et al., 2014). These cells have been extensively characterized previously in our laboratory (Gatto et al., 2021; Meyer et al., 2014).

**Figure 5:**
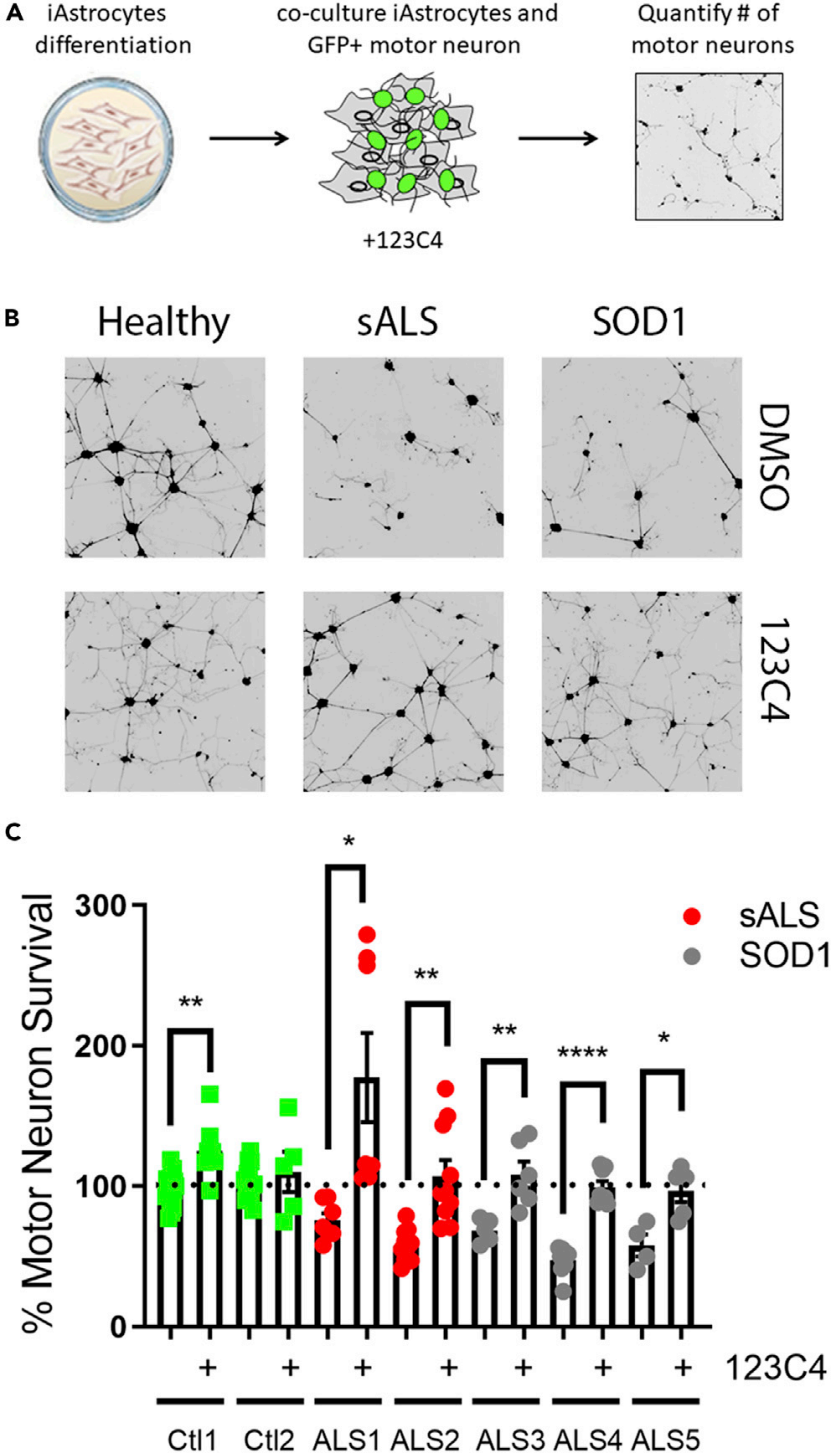
EphA4 agonist 123C4 protects from iAstrocyte-mediated motor neuron death (A) schematic of iAs:motor neuron compound screen. (B) Representative images of motor neurons (black) co-cultured with iAstrocytes. (C) Quantitation of motor neuron survival. Quantification of motor neuron survival. Only motor neurons with neurites exceeding 50 microns were counted. Data represent three independent experiments. 123C4 was added at 100 μM concentration at the time of motor neuron seeding. 123C4 was not removed from co-culture. Statistical analysis was performed using Student’s *t* test comparing cells in treated and untreated conditions (line) (∗p < 0.05, ∗∗p < 0.005, ∗∗∗p < 0.0005, ∗∗∗∗p < 0.0001). The black signal in the images of motor neurons represents the Hb9-GFP.

Here, we tested the effectiveness of synthetic agonists by co-culturing patient iAs (2 sALS and 3 mtSOD1, Sup. Table 1) and mouse motor neurons in the presence of 123C4 (Figure 5A). Three days following co-culture surviving motor neurons were imaged (Figure 5B). As previously described, both sALS and mutant SOD iAs were toxic to mouse motor neurons. Importantly, the addition of ephrin agonist, 123C4 prevented iAs-mediated motor neuron toxicity (Figures 5B and 5C) for both iAs from patients with sALS and mutant SOD1. These findings suggest that Ephrin 123C4 agonist can effectively prevent motor neuron death in multiple ALS patient subpopulations.

We have previously reported that 123C4 acts on neuronal EphA4 (Wu et al., 2017; Baggio et al., 2021). We found that following 123C4 binding to this neuronal receptor, EphA4 is internalized within the neurons. Some physiological of this internalization include the phosphorylation of EphA4 and the collapse of cortical growth cones. ^24, 28^ In this current article, we are evaluating the effects of 123C4 and new generation ligands on preventing astrocyte-mediated neurotoxicity, which presumably occurs through astrocyte ephrinB2 ligand binding to neuronal EphA4 receptors. Our hypothesis is that binding of 123C4 to neuronal EphA4 leads to its internalization, hence preventing the astrocyte ephrinB2 ligand from binding. Hence, to test this hypothesis we pretreated patient iAstrocytes with 123C4 prior to motor neuron addition (Figure 5A). At the time of motor neuron seeding, the drug was removed via aspiration, and motor neurons in freshly prepared media were cultured on the iAs monolayer. In addition, 123C4 was also added at the time of co-culture as described in Figure 5A.

**Figure 6:**
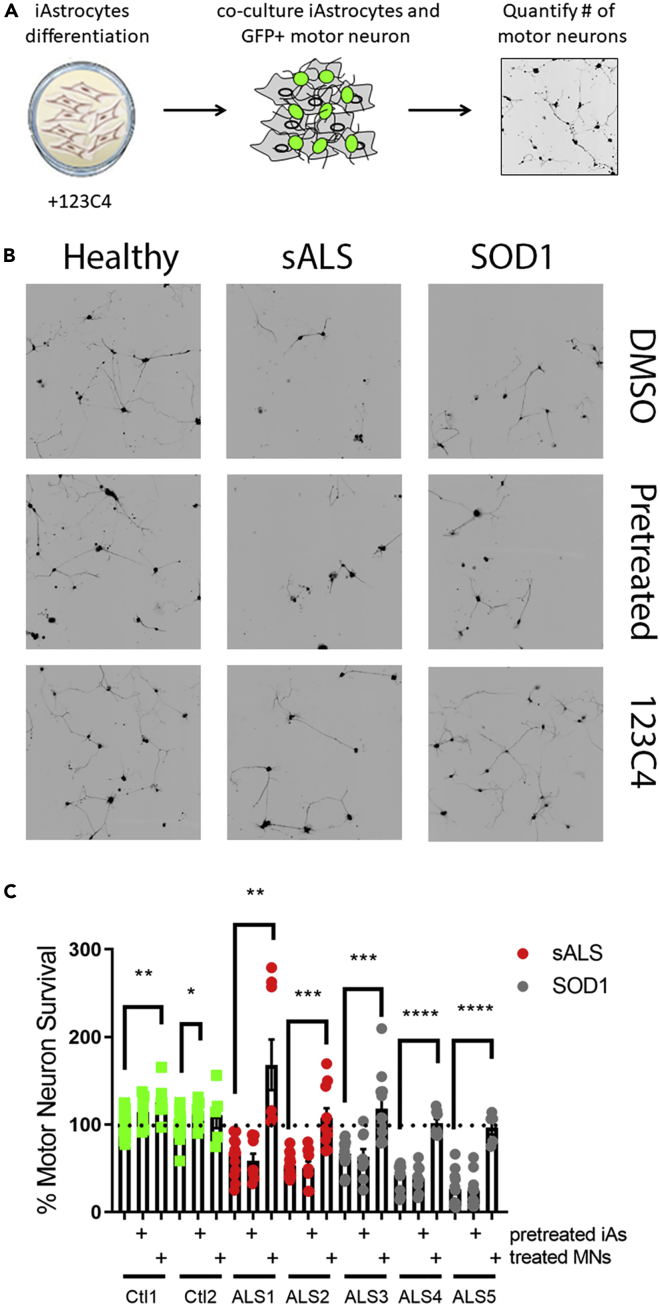
EphA4 agonist 123C4 pretreatment does not prevent iAstrocyte-mediated motor neuron death (A) schematic of iAs pretreatment compound screen. (B) Representative images of motor neurons (black) co-cultured with iAstrocytes. (C) Quantification of motor neuron survival. Only motor neurons with neurites exceeding 50 microns were counted. Data represent three independent experiments. Statistical analysis was performed using Welch’s unpaired t-test comparing different cell treatment groups to the untreated conditions (line, ∗p < 0.05, ∗∗p < 0.005, ∗∗∗p < 0.0005, ∗∗∗∗p < 0.0001). The black signal in the images of motor neurons represents the Hb9-GFP.

To accommodate a compound screen of this size, co-culture assays were adapted to a 384 well format. Interestingly, pretreatment of patient iAs with 123C4 did not prevent motor neuron death in the presence of ALS astrocytes (Figures 6B and 6C). In this assay, motor neuron morphology was slightly changed owing to being seeded in a smaller surface area. However, motor neuron survival was not impacted by this change. Importantly, addition of 123C4 at the time of motor neuron seeding prevented iAs-mediated motor neurons’ toxicity for all lines tested (Figures 6B and 6C). Combined, this data suggests that the EphA4 agonistic agents can prevent iAs-mediated motor neuron death. Although the detailed mechanism of this observed neuroprotection remains to be elucidated, our data suggested that EphA4 agonistic agents can alter ephrin-mediated interactions between astrocytes and motor neurons.

**Figure 7:**
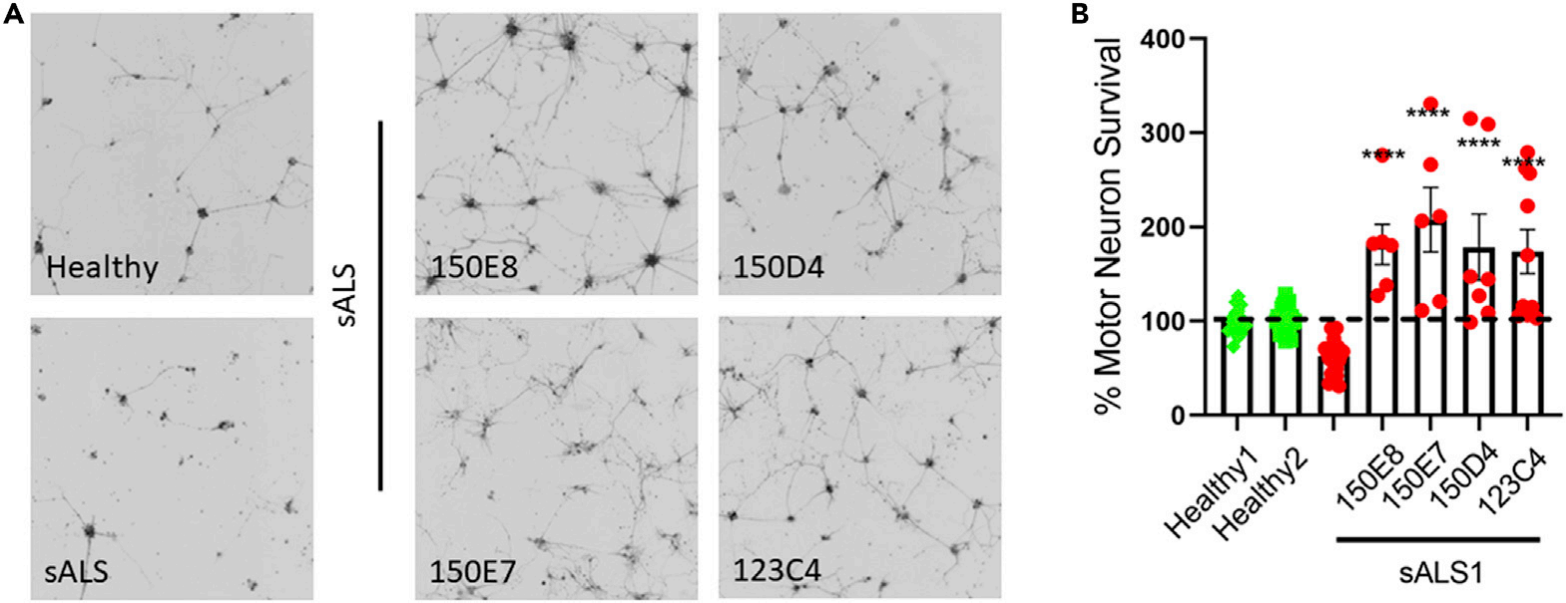
EphA4 agonistic agents prevent iAstrocyte-mediated motor neuron death at significantly lower concentrations (A) Representative images of motor neurons (black) co-cultured with iAstrocytes. (B) Quantification of motor neuron survival. Only motor neurons with neurites exceeding 50 microns were counted. Data represent three independent experiments. 123C4 was added at 100 μM concentration; 150E8, 150E7, and 150D4 were administered at 10 μM. Statistical analysis was performed using a one-way ANOVA compared to untreated ALS1 followed by Dunnet’s post-hoc analysis (∗p < 0.05, ∗∗p < 0.005, ∗∗∗p < 0.0005, ∗∗∗∗p < 0.0001). The black signal in the images of motor neurons represents the Hb9-GFP.

Following these promising preliminary results more potent and selective EphA4 agonists, 150E7, 150E8, and 150D4 were developed (Baggio et al., 2021). These compounds were tested in the co-culture assay at 10 μM, hence at a concentration that is 10-fold lower than what is effective for 123C4. Interestingly, all three derivatives were effective at preventing motor neuron death at this lower dose in one patient cell lines (Figures 7A-7C) and the same effect was observed in a second patient line in which the experiment was replicated two times (Figure S3). This data suggests that these newer and more potent agents have a lower therapeutic threshold than the original compound. In addition, these compounds are more soluble than 123C4 (Table 1) which facilitates both IV and intrathecal delivery methods.

## Discussion

Current studies evaluating the therapeutic potential of EphA4 antagonists or agonists have been performed to date in either mouse or zebrafish models with mutations in the superoxide dismutase gene, that is representing only one of many genes known to cause ALS. Consequently, it is difficult to directly translate the findings in these model systems directly to human patients as the heterogeneity of the ALS population is not effectively represented. Furthermore, studies using only the SOD1 mouse model have provided conflicting results on EphA4 receptor modulation, either genetically or by using small molecule antagonists or ephrin mimetics. Collectively, however, data from various laboratories converge on the notion that unligated EphA4 may exacerbate neuronal cell death, and that this activity can be reversed by agonist binding, suggesting that the development of potent ephrin-mimetics represents a viable therapeutic option.

To test this hypothesis, we first scaled our co-culture assay up from a 96 well to a 384 well plate. Although we observed noticeable variation in our motor neurons cultured on a 384 well, we had no observable differences in motor neuron viability on our diseased lines. Furthermore, this scale-up had no effect on therapeutic response to 123C4. Subsequently, we demonstrated that 123C4 is therapeutically effective in the presence of both iAstrocytes and motor neurons. However, pretreatment of iAstrocytes has no therapeutic benefit suggesting that sole targeting of this cell type is not sufficient for a therapeutic effect. Hence, the 123C4 therapeutic mechanism of action is likely owing to either directly influencing the astrocyte: motor neuron ephrin receptor interactions or acting directly on the motor neuron EphA4 itself. This may explain why genetic modulation of the EphA4 receptor in an adult mouse was not as effective as in embryonic manipulation. Either way, the findings of this article suggest that earlier intervention, while the motor neuron receptor complexes are still targetable is crucial for therapeutic effect.

Here we show that 123C4 is therapeutically effective in preventing astrocyte mediate toxicity in sporadic and alternate mutant SOD1 patient backgrounds. This is particularly important as there is a lack of adequate model systems for patients with sporadic ALS. Thus, these findings suggest that the therapeutic potential of 123C4 is not limited to patients with SOD1 mutations hence it may also be effective for sALS. Perhaps more importantly, newer generation EphA4 agonists, 150E7, 150E8, and 150D4 are also effective in preventing sALS toxicity but at a lower effective dose that is closer to what is attainable *in vivo* (Baggio et al., 2021). This is particularly exciting as sALS populations represent 90% of total patients. The lower therapeutic dose for efficacy suggests these compounds would be key candidates to evaluate for further clinical development.

### Limitations of the study

Previous studies from our laboratory identified 123C4 as an EphA4 targeting agent that biding to EphA4-LBD caused phosphorylation and internalization of the receptor. More recently, we derived a second-generation ligand, namely 150D4, and studied in detail its interactions with EphA4-LBD using biophysical methods. In this current article, we tested the hypothesis that the treatment of MNs with 123C4, 150D4, or new 150D4-related agents reported here for the first time, could protect MNs from cell death induced by astrocytes derived from patients with ALS. We found that the pretreatment of ALS astrocytes with our agents did not protect MNs. On the contrary, significant protection was observed when the agents were added to the co-culture, suggesting that our agents present a neuroprotective effect by altering EphA4-signaling in MNs. Although our studies suggest that the ligands act as agonists with respect to EphA4 phosphorylation and suppression of growth cone collapse, the major limitation of our work is the lack of detailed cell mechanistic studies aiming at identifying the molecular and cell signaling events that are at the origin of the observed neuroprotection. Nonetheless, the unique EphA4 targeting agents reported in our article represent invaluable pharmacological tools to conduct these future studies in detail.

## STAR★Methods

### Key resources table

**Table:** 

REAGENT or RESOURCE	SOURCE	IDENTIFIER
**Antibodies**		

EphA4 Monoclonal Antibody (4C8H5)	Invitrogen	Cat# 37-1600
anti-phosphotyrosine antibody	BD Transduction	Cat# #610000
HRP-conjugated anti-mouse secondary antibodies	Jackson ImmunoResearch	Cat# 715-035-150
goat anti-human IgG	Jackson ImmunoResearch,	Cat# 109-005-003

**Chemicals, peptides, and recombinant proteins**		

BAL Resin	CreoSalus	Cat# SA5070
4-Morpholinoanilin	Millipore Sigma	Cat# 197157-5G
1-Boc-4-(4′-aminophenyl)piperazine	ChemImpex	Cat# 24311
Fmoc-Gly-OH	Millipore Sigma	Cat# 47627-250G-F
Fmoc-D-Ala-OH	Novabiochem	Cat# 8.52142
Fmoc-L-HomoArg(Pbf)-OH	ChemImpex	Cat# 14077
Fmoc-L-4,4′-biphenylalanine	Combi-Blocks	Cat# SS-0620
Fmoc-5-Hydroxy-L-tryptophan	Acrotein	Cat# A-2534
(1S, 3S)-3-(Boc)Aminocyclohexane-1-Carboxylic Acid	Astatech	Cat# P15223
Sodium triacetoxyborohydride, 95%	Alfa Aesar	Cat# B22060
protein-A agarose beads	Sigma	Cat# P1406
reducing sample buffer	Sigma	Cat# S3401
Tris-glycine SDS-PAGE pre-cast gel	Invitrogen	Cat# XP08160BOX
ECL Detection reagent	Thermo Scientific,	Cat# 32106
ephrinA1-Fc	R&D Systems	CAt# 602-A1
protease inhibitor cocktail	Sigma	Cat# P8340

**Experimental models: Cell lines and cell culture**		

FGF	preprotech	100-18B
DMEM/F12	Gibco	10565042
EmbryoMax DMEM	Millipore	SLM-220-B
Knock-out DMEM	Invitrogen	10829-018
MN isolated from spinal cords of B6.Cg-Tg(Hlxb9-GFP)1Tmj/J (Hb9-GFP) mice	The Jackson Laboratory	Strain #:005029
DMEM, High Glucose	Gibco	10569010
F12 (ham)	ThermoFisher	11765–062
BDNF	ThermoFisher	PHC7074
CDNF	ThermoFisher	PHC7015
GDNF	ThermoFisher	PHC7041
SAG	Sigma	56660-1MG
Retinoic Acid	Sigma	R2625
LIF (human)	Millipore	ESG1106
Fibronectin	Millipore	FC010-10MG
Mouse Embryonic Feeders	Millipore	PMEF-CF
Glucose	ThermoFisher	D16-1
N2	Gibco	17502048
B27	Invitrogen	17504044
L-Glutamine	Gibco	25030–081
NEAA	Gibco	11140–50
Knockout serum	Gibco	10828–028
ES FBS	Gibco	10439–024
Postmortem astrocytes and patients’ fibroblasts	Nationwide Children’s Hospital	
Fetal Bovine Serum, certified	Gibco	16000–036

**Software and algorithms**		

Bruker TopSpin 3.6.1 NMR Software	Bruker	www.bruker.com
ChromNAV 2.0 HPLC Software	JASCO	https://jascoinc.com/products/chromatography/hplc/hplc-software/
ITCRun 3.8	TA Instrument	https://www.tainstruments.com/support/software-downloads-support/downloads/
NanoAnalyze 3.10	TA Instrument	https://www.tainstruments.com/support/software-downloads-support/downloads/
BD Influx sorter using *sortware* software	BD biosciences	https://www.bd.com/resource.aspx?IDX=17866

### Resource availability

#### Lead contact

Information and requests for resources and reagents should be directed to and will fulfilled by Dr. Maurizio Pellecchia (Maurizio.Pellecchia@ucr.edu).

#### Materials availability

The EphA4 targeting agents generated in this study will be made available on request, for research purposes, but we may require a small payment and a completed materials transfer agreement.

### Experimental model and subject details

#### Primary motor neuron cultures

Primary motor neurons were isolated as previously described (Wang et al., 2017) with minor modifications as reported here. Briefly, cells were isolated from spinal cords of B6.Cg-Tg(Hlxb9-GFP)1Tmj/J (Hb9-GFP) mice at postnatal day (P) 0–2. Tissues were dissected, cut into 1–2 mm pieces, and treated with a papain/DNase I (0.1 M PBS/0.1% BSA/25 mM glucose/5% papain/1×DNase I [Sigma, #D5025-15K]) solution for 20 min at 37C. Cells were mechanically dissociated, filtered using a 100 μm cell strainer and further purified using OptiPrep gradient centrifugation. Neurons were plated on poly-D-lysine (0.5 mg/mL) and laminin (5 μg/mL) 6-well plates (350,000 per well of 6-well plate) in Neurobasal media with 25 mM glutamine, 1% penicillin-streptomycin, B27 supplement (Invitrogen, #17504-044). After 2 h media was changed to a fresh media in addition containing 5% horse serum (Gibco, #26050-070) and 10 ng CTNF (Sino Biological, #11841-H-07 × 10^−5^). Cells were maintained under 5% CO_2_/10% O_2_ atmosphere at 37°C.

#### Patient-derived astrocytes

Postmortem astrocytes were isolated and expanded as previously described (Meyer et al., 2014). Cells were cultured in astrocyte medium (DMEM gluatmax) containing 10% FBS and 0.2% N2. 10,000 astrocytes were seeded into 96 well plates for co-culture.

Patient skin punches were obtained and maintained in culture until fibroblast were extracted. Patient fibroblasts were reprogrammed directly into neuronal progenitor cells (NPCs) as previously described (Dennys et al., 2021). Induced Astrocytes were generated by seeding a low quantity of NPCs into astrocyte medium (DMEM media containing 10% FBS and 0.2% N2) for five days. Following differentiation, iAstrocytes were lifted and seeded into a 96 well (10,000 cells per well) or 384 well (2500 cells per well) plate. Differentiations were repeated at separate passages and each differentiation was counted as an independent experiment.

#### GFP + motor neurons

Motor neurons expressing GFP under an HB9 promotor were differentiated from mouse embryonic bodies as previously described (Dennys et al., 2021) for each independent experiment, and subsequently generated EBs were dissociated and sorted using Becton-Dickinson Influx sorter using *sortware* software. Cells are sorted through a 100 μm tip with sheath pressure of 27. GFP + motor neurons were seeded in a 96 well plate (10,000 cells per well) or 384 well (1,000 cells per well) in triplicate for each condition. Co-cultures were imaged with InCell Analyzer 6000 automated microscopy system for up to six days. Automated data analysis was conducted using InCell developer and analyzer software, with live motor neurons being defined as neurons with neurite outgrowth of greater than 50 μm. For each independent experiment the motor neuron count is normalized to the average of the healthy controls to give % survival. Normalized counts are then pooled amongst all replicate experiments and a student T-test was utilized to compare treated lines to their corresponding untreated controls.

### Method details

#### Synthetic chemistry

All reagents and solvents were obtained from commercial sources, including the Fmoc-protected amino acids and resins for solid phase synthesis. The concentration of stock solutions was evaluated by NMR spectroscopy recorded on Bruker Avance III 700 MHz equipped with a TCI cryo-probe. High resolution mass spectral data were acquired on an Agilent 6545 Q-TOF LC/MS instrument. RP-HPLC purifications were performed on a JASCO preparative system equipped with a PDA detector and a fraction collector controlled by a ChromNAV system (JASCO) on a XTerra C18 10μm 10 × 250 mm (Waters). Purity of tested compounds was assessed by HPLC using an Atlantis T3 3 μm 4.6 × 150 mm^2^ column (H_2_O/acetonitrile gradient from 5 to 100% in 45 min). All compounds have a purity >95% (Figures S1 and S2). All agents were synthesized in house by standard solid-phase Fmoc peptide synthesis protocols on BAL resin (Figures S1 and S2). Briefly, were used 3 equiv of Fmoc-AA, 3 equiv of HATU, 3 equiv of OximaPure, and 5 equiv of DIPEA in 1 mL of DMF for each coupling reaction. The coupling reaction was allowed to proceed for 1 h. Fmoc deprotection was performed by treating the resin-bound peptide with 20% piperidine in DMF twice. Peptides were cleaved from Rink amide resin with a cleavage cocktail containing TFA/TIS/water/phenol (94:2:2:2) for 5 h. The cleaving solution was filtered from the resin, evaporated under reduced pressure and the peptides precipitated in Et_2_O, centrifuged and dried in high vacuum.

(***150E7)*** *(1S,3S)-N-((S)-1-(((S)-3-([1,1'-biphenyl]-4-yl)-1-(((S)-6-guanidino-1-(((R)-1-((4-morpholinophenyl)amino)-1-oxopropan-2-yl)amino)-1-oxohexan-2-yl)amino)-1-oxopropan-2-yl)amino)-3-(5-hydroxy-1H-indol-3-yl)-1-oxopropan-2-yl)-3-aminocyclohexane-1-carboxamide.* BAL resin was used as solid-phase support (0.05 mmol scale). A BAL resin was loaded using a solution of 4-(Morpholinomethyl)aniline (3 eq.) in DMF added to the reactor and shaken for 30 min, followed by reduction using Sodium triacetoxyborohydride (3 eq., overnight reaction at room temperature). The resin was subsequently filtered, washed with DMF (3x), with DCM (3x) and again three times with DMF. For the coupling of Fmoc-D-Alanine on the secondary amine, reaction time was increased to 2 h. Fmoc deprotection and peptide elongation then followed standard procedures described in the general chemistry section (Figure S1). After cleavage, the crude was purified by preparative RP-HPLC using a XTerra C18 (Waters) and water/acetonitrile gradient (5% to 100%) containing 0.1% TFA. HRMS: calcd 969.5225 (M); obs 970.5310 (M + H)^+^, 992.5093 (M + Na)^+^.

(***150E8)*** *(1S,3S)-N-((S)-1-(((S)-3-([1,1'-biphenyl]-4-yl)-1-(((S)-6-guanidino-1-oxo-1-((2-oxo-2-((4-(piperazin-1-yl)phenyl)amino)ethyl)amino)hexan-2-yl)amino)-1-oxopropan-2-yl)amino)-3-(5-hydroxy-1H-indol-3-yl)-1-oxopropan-2-yl)-3-aminocyclohexane-1-carboxamide.* BAL resin was used as solid-phase support (0.05 mmol scale). A BAL resin was loaded using a solution of 1-Boc-4-(4-aminophenyl)piperazine (3 eq.) in DMF added to the reactor and shaken for 30 min, followed by reduction using Sodium triacetoxyborohydride (3 eq., overnight reaction at room temperature). The resin was subsequently filtered, washed with DMF (3x), with DCM (3x) and again three times with DMF. For the coupling of Fmoc-Glycine on the secondary amine, reaction time was increased to 2 h. Fmoc deprotection and peptide elongation then followed standard procedures described in the general chemistry section (Figure S2). After cleavage, the crude was purified by preparative RP-HPLC using a XTerra C18 (Waters) and water/acetonitrile gradient (5%100%) containing 0.1% TFA. HRMS: calcd 954.5228 (M); obs 955.5319 (M + H)^+^.

#### NMR spectroscopy and ITC measurements

NMR spectra were acquired on Bruker Avance III 700MHz spectrometer equipped with a TCI cryoprobe. All NMR data were processed and analyzed using TOPSPIN 3.6.1 (Bruker, Billerica, MA, USA). 2D-[^13^C,^1^H]-HSQC experiments were acquired with 20 μM proteins using 16 scans with 2,048 and 256 complex data points in the ^1^H and ^15^N dimensions, respectively, at 298 K. Chemical shift changes (Δδ) in the 2D [^13^C, ^1^H] spectra were calculated as weight average perturbations observed in the ^1^H and ^13^C dimensions using the following Equation:

$$\text{Δ}\delta =\sqrt{\frac{1}{2}*\left[{\left({\text{Δ}}^{1}H\right)}^{2}+{\left(0.3*{\text{Δ}}^{13}C\right)}^{2}\right]}$$

Isothermal titration calorimetry measurements were performed using the Affinity ITC Autosampler from TA Instruments (New Castle, DE). The titrations were performed in a reverse fashion by titrating the protein into the ligand solution. All the measurements were performed at 25°C dissolving the agents in 25 mM Tris at pH 7.5, 150 mM NaCl, and a final DMSO concentration of 1%. The syringe was filled with a 200 μM solution of EphA4-LBD, EphA3-LBD Chimera, or EphA2-LBD, and 20 injections of 2.5 μL each were performed into the cell containing a 30–40 μM solution of the compounds. The injections were made at 400 s intervals with a stirring speed of 75 rpm. All the solutions were kept in the autosampler at 4°C. Each titration was performed twice and individually using the ITCRun software, analyzed using NanoAnalyze software (TA Instruments, New Castle, DE) and subsequently exported into Microsoft Excel. K_d_ are reported as mean with standard error.

#### EphA4 receptor drug treatment

EphA4 receptor drug treatment was performed as previously described (Baggio et al., 2021) with minor modifications as reported here. Briefly, ephrinA1-Fc (R&D Systems, #602-A1) and human Fc (R&D Systems, #110-HG) were pre-clustered by the incubation with goat anti-human IgG (Jackson ImmunoResearch, #109-005-003) for 1 h at 4C. At 3 days *in vitro* (DIV) primary motor neurons were treated with pre-clustered Fc (2.5 μg/mL), ephrinA1-Fc (2.5 μg/mL), 10 μM 150D4, 1 and 10 μM 123C4, 150E7 and 150E8 for 15 min at 37C under 5% CO_2_/10% O_2_ atmosphere and then processed for western blotting analysis. 0.1% DMSO was used as a negative control.

#### Immunoprecipitation and western blot analysis

Immunoprecipitation and western blot analysis were performed similarly as we recently described (Baggio et al., 2021). Specifically, cells were collected and lysed in the lysis buffer (25 mM Tris-HCl, 150 mM NaCl, 5 mM EDTA, 1% Triton X-100, 1 mM sodium pervanadate, and protease inhibitor cocktail [1:100, Sigma, #P8340]) at 4C for 30 min. Cell lysates were cleared by centrifugation at 13,500 rpm for 20 min at 4C, then incubated with protein-A agarose beads (Sigma, #P1406) and anti-EphA4 antibody (Invitrogen, #371600), for 2 h at 4C. Beads and cell lysate were boiled in reducing sample buffer (Laemmli 2× concentrate, Sigma, #S3401). Samples were briefly spun down and the supernatant was run on an 8–16% Tris-glycine SDS-PAGE pre-cast gel (Invitrogen, #XP08160BOX). Proteins were transferred onto a Protran BA 85 nitrocellulose membrane (GE Healthcare) and blocked for 1 h at room temperature in 5% BSA. The blots were incubated with anti-phosphotyrosine antibody (BD Transduction, #610000) in Tris-buffered saline (TBS)/0.1% Tween 20/1% BSA at 4C overnight. Membranes then were washed 3 × 10 min with TBS/0.1% Tween 20/1% BSA and incubated with HRP-conjugated anti-mouse secondary antibodies at 1:5000 (Jackson ImmunoResearch, #715-035-150) for 2 h at room temperature in a TBS/0.1% Tween 20/1% BSA solution. Blots were further incubated with ECL Detection reagent (Thermo Scientific, #32106) and imaged using ChemiDoc imaging system (Bio-Rad). For reprobing, membrane blots were washed in stripping buffer (2% SDS, 100 mM β-mercaptoethanol, 50 mM Tris-HCl [pH 6.8]) for 30 min at 55C and then washed 5 × 5 min with TBST, blocked with 5% skim milk, and re-probed for EphA4 (Invitrogen, #371600). Blots were washed 3 × 10 min with TBS/0.1% Tween 20 and then incubated with anti-mouse HRP-conjugated secondary antibodies in TBS/0.1% Tween 20/1% BSA (Jackson ImmunoResearch, #715-035-150) for 2 h at room temperature. After the incubation, blots were washed 3 × 10 min with TBS/0.1% Tween 20 and developed as described earlier. Band density was analyzed by measuring band and background intensity using Adobe Photoshop CS5.1 software. ChaT expression in cell lysate was tested by immunoblot as described above to confirm identity of motor neurons for each culture.

### Quantification and statistical analysis

GraphPad Prism 6.0 was used for data and statistical analyses. Generally, t-test analysis was performed comparing treated cells to their corresponding untreated controls, and p values <0.05 were considered as statistically significant, indicated in the captions to Figure 3, Figure 4, Figure 5, Figure 6, Figure 7. As indicated in the captions to Figures 3, 6, and 7, statistical analysis was also performed using one-way ANOVA followed by Bonferroni’s or by Dunnet’s post-hoc analyses. Dissociation constants data of the reported agents in table for EphA4-LBD represent mean values ±SE, from two isothermal titration calorimetry independent measurements.

## Data Availability

• Any raw or processed data reported in this paper is available from the lead contact upon request. • This paper does not report original code. • Any additional information required to reanalyze the data reported in this work paper is available from the lead contact upon request
