# Supplementary material for: EphA4 targeting agents protect motor neurons from cell death induced by amyotrophic lateral sclerosis -astrocytes
Source: iScience. 2022 Aug 5;25(9):104877. doi: 10.1016/j.isci.2022.104877 (PMC9404653; doi:10.1016/j.isci.2022.104877)
Supplement: Document S1. Figures S1–S4 and Table S1 [file mmc1.pdf]

iScience, Volume *25*

Supplemental information

EphA4 targeting agents protect motor neurons from cell death induced by amyotrophic lateral sclerosis -astrocytes

Cassandra Dennys, Carlo Baggio, Rochelle Rodrigo, Florence Roussel, Anna Kulinich, Sarah Heintzman, Ashley Fox, Stephen J. Kolb, Pamela J. Shaw, Iryna M. Ethell, Maurizio Pellecchia, and Kathrin C. Meyer

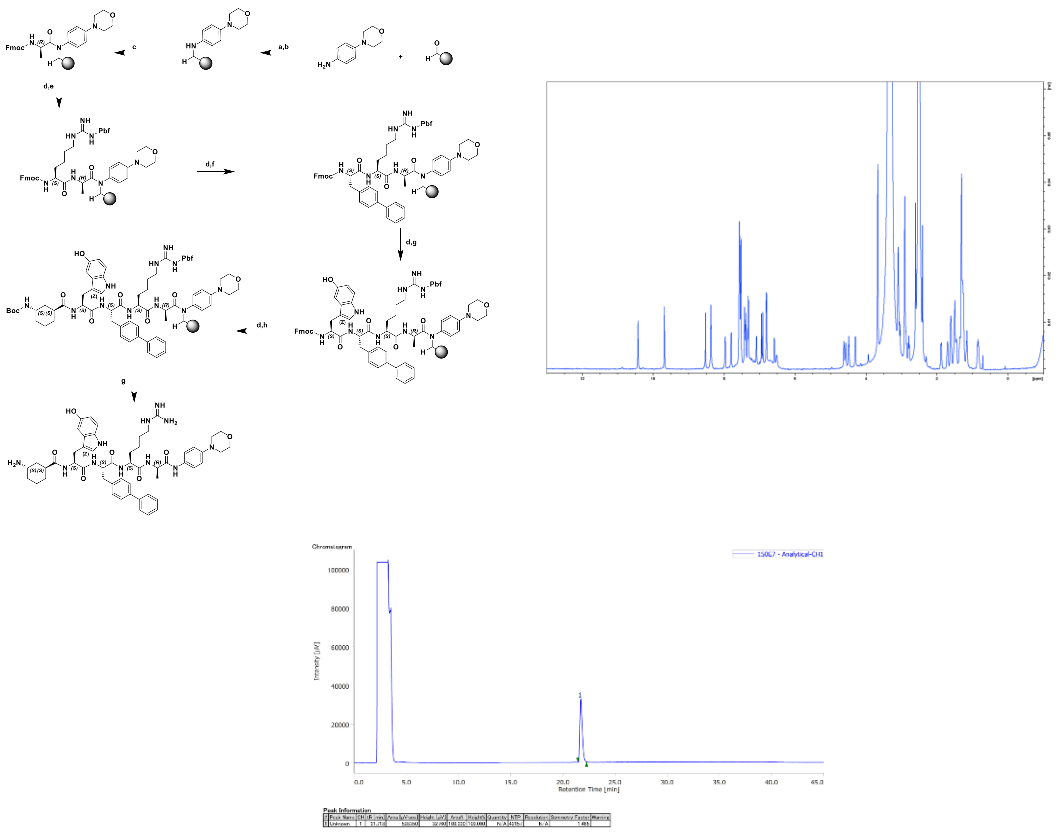

Figure S1. **General scheme for the synthesis of compounds 150E7 and its characterization** (related to Table 1). *Top left*: Conditions: (a) DMF, 30 min, rt; (b) sodium triacetoxyborohydride (3 eq.), o/n, rt; (c) Fmoc-D-Ala-COOH (3 equiv), HATU (3 eq.), Oxyma Pure (3 eq.), DIPEA (5 eq.), 2 h, rt; (d) 20% 4-methylpiperidine in DMF, rt; (e) Fmoc-L-HomoArg(Pbf)-OH (3 eq.), HATU (3 eq.), Oxyma Pure (3 eq.), DIPEA (5 eq.), 1 h, rt; (f) Fmoc-p-phenyl-L-phenylalanine (3 eq.), HATU (3 eq.), Oxyma Pure (3 eq.), DIPEA (5 eq.), 1 h, rt; (g) Fmoc-5-Hydroxy-L-tryptophan (3 eq.), HATU (3 eq.), Oxyma Pure (3 eq.), DIPEA (5 eq.), 1 h, rt; (h) (1S,3S)-3-(Boc-amino)cyclohexanecarboxylic acid (3 eq.), HATU (3 eq.), Oxyma Pure (3 eq.), DIPEA (5 eq.), 1 h, rt; (g) TFA/TIS/water/phenol (94:2:2:2), 5 h, rt. *Top, right:*1D ^{1}H NMR spectra recorded in d6-DMSO on Bruker Avance III 700 MHz equipped with a TCI cryo-probe. *Bottom: HPLC trace*. Analytical run was accomplished using Atlantis T3 3μm 4.6x150 mm (H_2O/ACN gradient from 5% to 100% in 45 min). Purity > 95%.

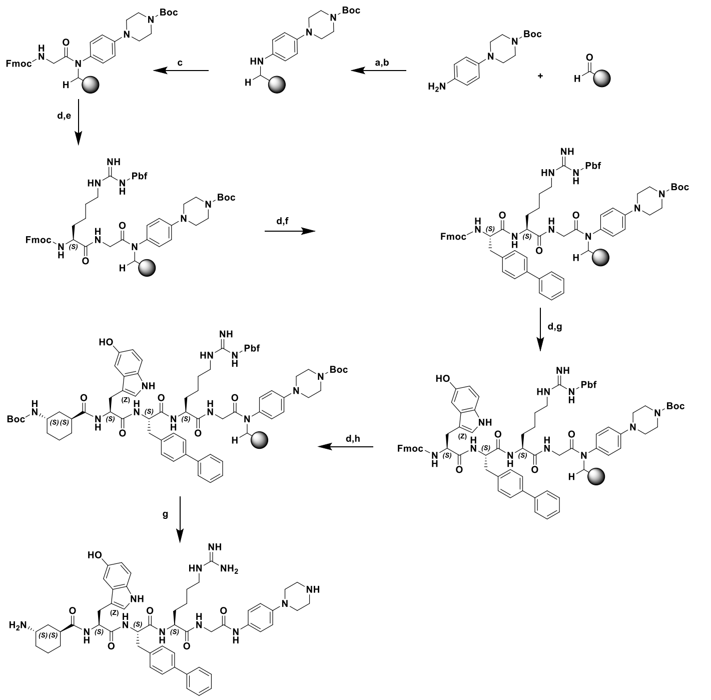

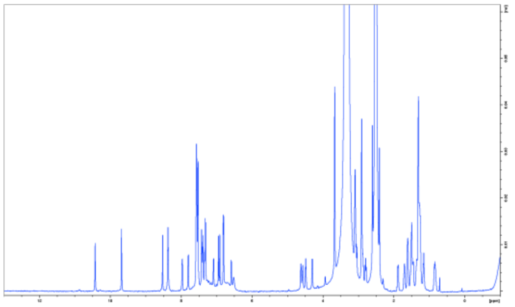

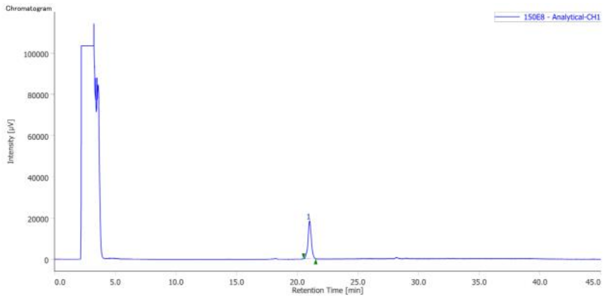

Peak Information

#	Peak Name	CH	tR [min]	Area [µV·sec]	Height [µV]	Area%	Height%	Quantity	NTP	Resolution	Symmetry Factor	Warning
1	Unknown	1	21.015	349261	18442	100.000	100.000	N/A	31194	N/A	0.988	

Figure S2. **General scheme for the synthesis of compounds 150E8 and its characterization** (related to Table 1). *Top left*: Conditions: (a) DMF, 30 min, rt; (b) sodium triacetoxyborohydride (3 eq.), o/n, rt; (c) Fmoc-Gly-COOH (3 equiv), HATU (3 eq.), Oxyma Pure (3 eq.), DIPEA (5 eq.), 2 h, rt; (d) 20% 4-methylpiperidine in DMF, rt; (e) Fmoc-L-HomoArg(Pbf)-OH (3 eq.), HATU (3 eq.), Oxyma Pure (3 eq.), DIPEA (5 eq.), 1 h, rt; (f) Fmoc-p-phenyl-L-phenylalanine or Fmoc-4-(2-methoxyphenyl)-L-phenylalanine (3 eq.), HATU (3 eq.), Oxyma Pure (3 eq.), DIPEA (5 eq.), 1 h, rt; (g) Fmoc-5-Hydroxy-L-tryptophan (3 eq.), HATU (3 eq.), Oxyma Pure (3 eq.), DIPEA (5 eq.), 1 h, rt; (h) (1S,3S)-3-(Boc-amino)cyclohexanecarboxylic acid (3 eq.), HATU (3 eq.), Oxyma Pure (3 eq.), DIPEA (5 eq.), 1 h, rt; (g) TFA/TIS/water/phenol (94:2:2:2), 5 h, rt. *Top right:* 1D ^{1}H NMR spectra recorded in d6-DMSO on Bruker Avance III 700 MHz equipped with a TCI cryo-probe. *Bottom: HPLC trace.* Analytical run was accomplished using Atlantis T3 3µm 4.6x150 mm (H_2O/ACN gradient from 5% to 100% in 45 min). Purity > 95%.

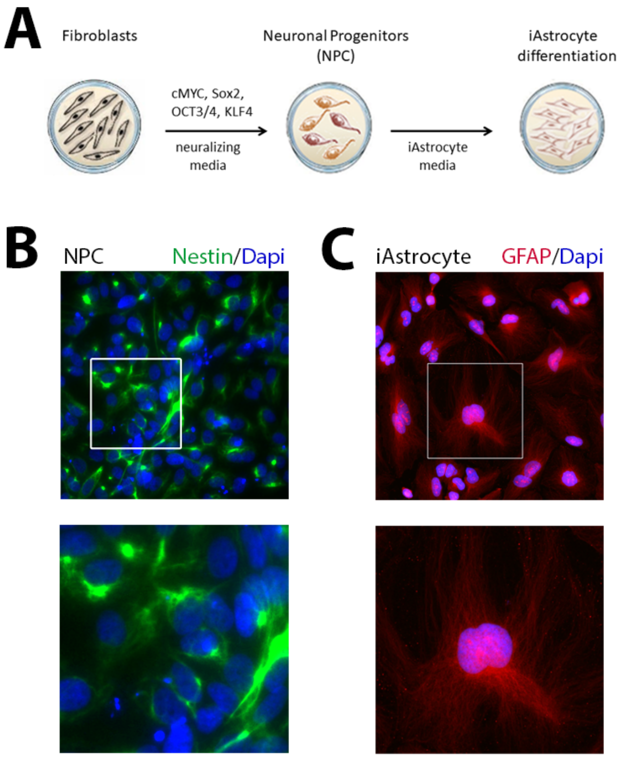

Figure S3: Patient fibroblasts can be reprogrammed directly into neuronal progenitor cells and subsequently differentiated into astrocytes (related to Figures 4-7). A) representative image of direct conversion protocol. B) Nesting (green) and Dapi (blue) immunostaining of neuronal progenitor cells. C) GFAP (red) and dapi (blue) staining of induced astrocytes.

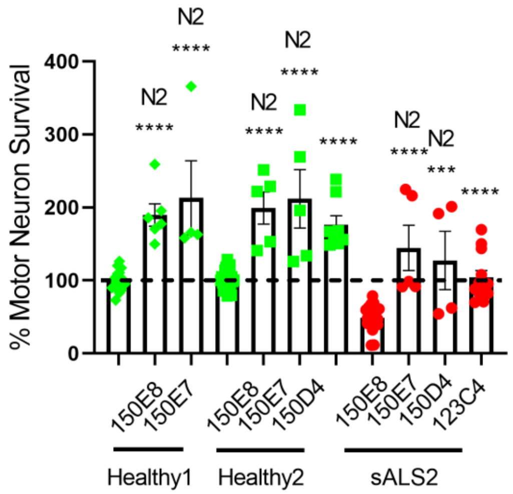

Figure S4: EphA4 agonistic agents prevent iAstrocyte mediated motor neuron death at significantly lower concentrations in a second sALS patient line (related to Figures 4-7). Quantification of motor neuron survival. Only motor neurons with neurites exceeding 50 microns were counted. Data represents 2-3 independent experiments. 123C4 was added at 100 μM concentration; 150E8, 150E7, and 150D4 were administered at 10 μM. Statistical analysis was preformed using student t-test comparing drug treatments against untreated sALS cells.

ID	Age	Sex	Classification	Generation
Ctl1	64	male	Healthy	corriel: AG08125
Ctl2	unknown adult	male	Healthy	Sheffield
ALS1	29	male	sALS	OSU
ALS2	55	female	sALS	OSU
ALS3	56	male	SOD1	corriel: ND29509
ALS4	63	female	SOD1	OSU
ALS5	40	male	SOD1	Sheffield

Table S1: Description of cell lines utilized (related to figures 4-7).
